# Trace phase formation, crystallization kinetics and crystallographic evolution of a lithium disilicate glass probed by synchrotron XRD technique

**DOI:** 10.1038/srep09159

**Published:** 2015-03-17

**Authors:** Saifang Huang, Zhaohui Huang, Wei Gao, Peng Cao

**Affiliations:** 1 Department of Chemical & Materials Engineering, the University of Auckland, PB 92019, Auckland 1142, New Zealand; 2 School of Materials Science and Technology, China University of Geosciences (Beijing), Beijing 100083, P. R. China

## Abstract

X-ray diffraction technique using a laboratory radiation has generally shown limitation in detectability. In this work, we investigated the *in situ* high-temperature crystallization of a lithium disilicate glass-ceramic in the SiO_2_–Li_2_O–CaO–P_2_O_5_–ZrO_2_ system with the aid of synchrotron radiation. The formation of lithium metasilicate and other intermediate phases in trace amount was successfully observed by synchrotron X-ray diffraction (SXRD). The crystallization mechanism in this glass was thus intrinsically revised to be the co-nucleation of lithium metasilicate and disilicate, instead of the nucleation of lithium disilicate only. The phase content, crystallite size and crystallographic evolutions of Li_2_Si_2_O_5_ in the glass-ceramic as a function of annealing temperature were studied by performing Rietveld refinements. It is found that the growth of Li_2_Si_2_O_5_ is constrained by Li_2_SiO_3_ phase at 580–700°C. The relationship between the crystallographic evolution and phase transition was discussed, suggesting a common phenomenon of structural response of Li_2_Si_2_O_5_ along its *c* axis to other silicon-related phases during glass crystallization.

Glass science and technology has been fundamentally important to the development of glasses and glass-ceramics over decades. The kinetics and thermodynamics of nucleation are described by several theories including the classical nucleation theory (CNT) and Johnson–Mehl–Avrami–Kolmogorov (JMAK) theory^1^. Traditionally, thermoanalytic techniques, such as differential scanning calorimetry (DSC) or differential thermal analysis (DTA), have been used to study the non-isothermal crystallization kinetics^2^,^3^,^4^. Recently, we have successfully applied the state-of-the-art synchrotron radiation to study the mechanism, kinetics and crystallographic change during the crystallization of lithium disilicate glasses^5^,^6^,^7^.

It has been well known that there are three types of reaction sequence occurring in the fabrication of lithium disilicate glass-ceramics, depending on glass composition^5^. One (type I) is the simultaneous nucleation of lithium metasilicate (LS) and lithium disilicate (LS_2_) phases, followed by the transformation of LS to LS_2_^7^,^8^,^9^. In the second type (type II), LS nucleates first and then transforms to LS_2_ at a higher temperature^10^. The third type (type III) of reaction sequence is that the LS_2_ phase directly forms in the glasses whereas no LS phase forms^11^,^12^. The LS phase has very attractive machinability which enables the utilization of CAD/CAM technology for the fabrication of dental products with complex shapes. Thus glasses with types I and II reaction sequences are preferred for dental applications^13^. Glasses of type III are however not very common in the literature, which is probably because that it is technologically not emphasized due to the absence of the intermediate phase.

In our previously study, we examined the phase transformation of a complex LS_2_ glass in the SiO_2_–Li_2_O–CaO–P_2_O_5_–ZrO_2_ glass system using both *in situ* and *ex situ* laboratory-based XRD techniques^14^. The glass-ceramics derived from this composition have an optimized flexural strength of 439 ± 93 MPa. The crystallization mechanism was deemed to be the direct precipitation of LS_2_ phase (type III) whereas no LS phase was detected during glass crystallization^14^. In this study, we revisited the high-temperature phase transformation of the same LS_2_ glass using synchrotron radiation. A trace amount of LS phase was identified at the initial nucleation stage as well as other intermediate phases during crystallization. The results from this study may shed insight on the understanding of crystallisation in glass-ceramics.

## Results

### Trace phase formation in the glass

**Figure 1:**
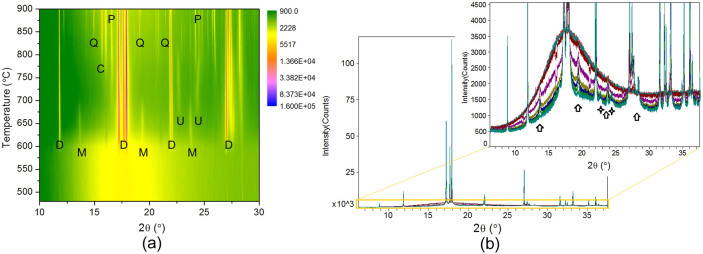
(a) *In situ* high-temperature XRD patterns (in color contour) of the complex lithium disilicate glass, (b) the line patterns of partial data to show the peaks of LS phase (directed by arrows) and peaks of other unidentified phase (directed by stars). D: lithium disilicate (LS_2_); M: lithium metasilicate (LS); P: lithium phosphate (LP); C: β-cristobalite (CR); Q: β-quartz (QZ); U: unidentified intermediate phase.

With the scheduled annealing profile, the *in situ* high-temperature XRD patterns of the complex lithium disilicate glass were acquired and depicted in a two-dimensional pattern in Figure 1a where the intensities are illustrated in color. No diffraction peaks were observed at temperatures below 580°C. There are five crystalline phases identified from the synchrotron diffraction patterns: LS, LS_2_, Li_3_PO_4_ (LP), β-cristobalite (CR), and β-quartz (QZ). The diffraction peaks initiated at 580°C can be assigned to LS and LS_2_ phases. The intensity of LS phase (~500 counts) is considerably lower than that of LS_2_ phase (~160,000 counts), as shown in Figure 1b. Such a high intensity ratio of LS_2_ to LS (over 300 times) may explain why the precipitation of LS in this glass was hardly detected by the conventional XRD technique^14^. Due to the distortion of the sample, data acquisition of synchrotron powder diffraction of this glass failed at a temperature ≥920°C where melting was accelerated.

### Kinetics of glass crystallization

**Figure 2:**
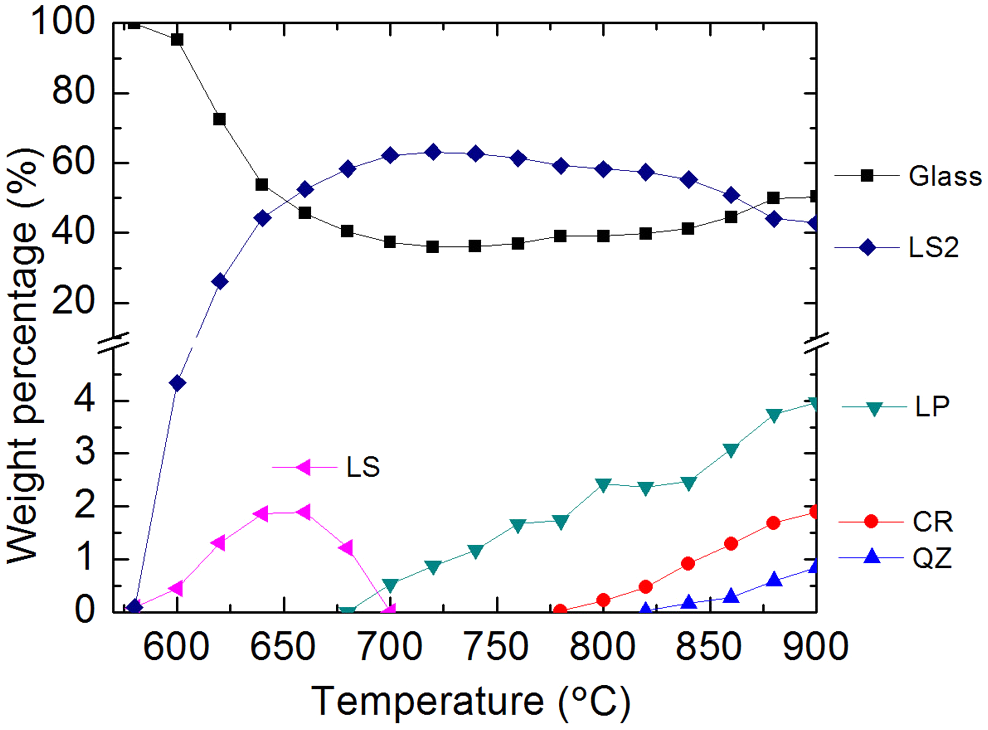
Weight fraction of phases in the glass-ceramics as a function of temperature. The quantitative results suggest a maximum of ~2 wt% LS precipitated from the glass.

The crystallographic parameters of LS_2_ and weight fraction of each phase are determined by applying Rietveld refinement using the full patterns. The change in weight fraction of involved phases during the glass crystallization is presented in Figure 2. Both lithium metasilicate and disilicate phases were detected at 580°C, with a limited quantity of 0.09 wt% and 0.08 wt% respectively as determined by quantitative phase analysis. The LS_2_ phase dominated the glass-ceramic during crystallization, which reached a fraction of above 50 wt% at 660°C. The crystallization of LS_2_ reached a maximum of ~63 wt% at ~760°C. In this glass, LS with a maximum amount of ~2 wt% formed and completely disappeared at 700°C.

With the P_2_O_5_ component added as a nucleating agent in the base glass, lithium phosphate (Li_3_PO_4_, LP) nuclei with short range order form in the glass at the early stage, which then trigger the compositional gradients of parent glass and lead to the nucleation of silicate phases^7^,^15^. In the current glass, the LP phase was observed at temperatures of 700°C and above. The silica phases including β-cristobalite and β-quartz crystallized when the temperature reached 780 and 820°C, respectively. As indicated in Figure 2, LP, CR and QZ showed a linear increasing trend after their precipitation. The complete crystallization of LP gave a maximum fraction of ~4 wt%, whereas the maximum fraction of CR and QZ was about 2 wt% and less than 1 wt%, respectively.

**Figure 3:**
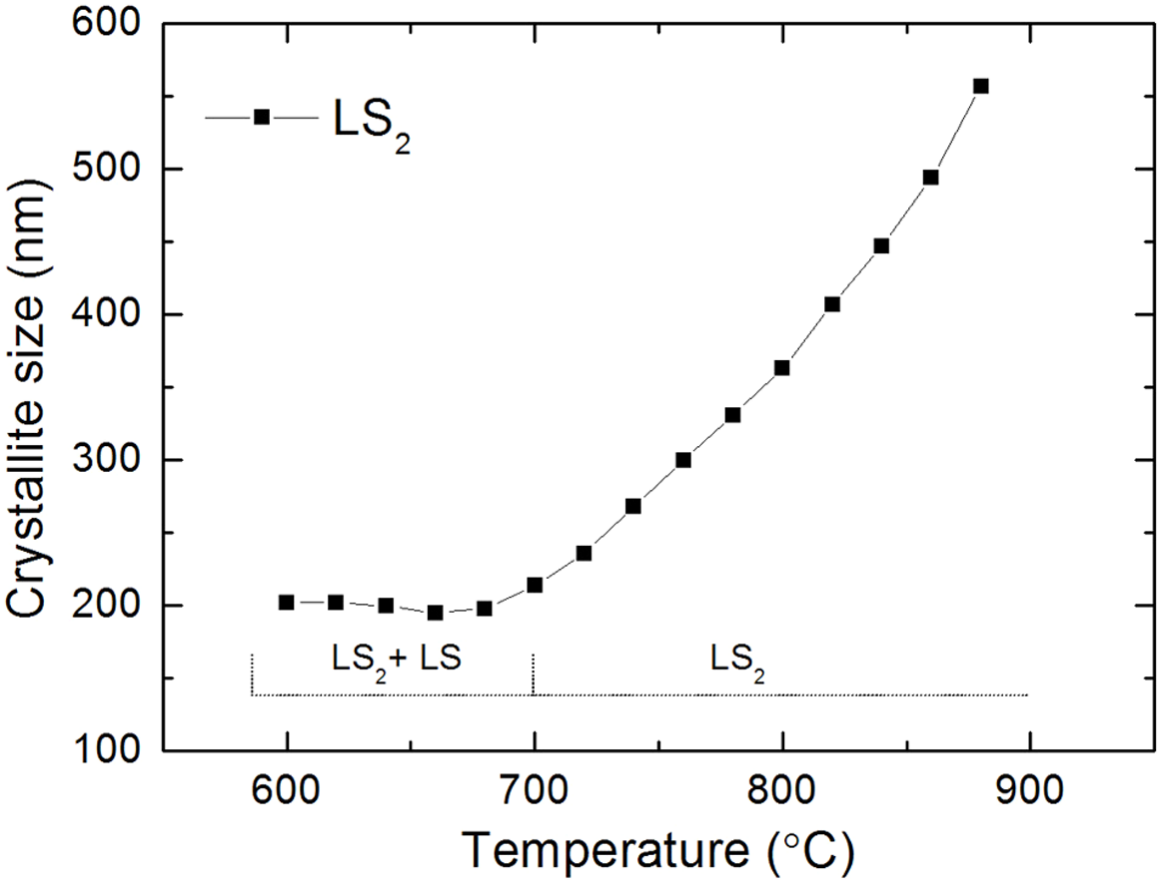
The crystallite size of LS_2_ phase as a function of temperature. The dotted line shows the silicate phase(s) existed in the corresponding temperature range. The crystal growth of LS_2_ is indicated to be hampered by the existence of LS.

The evolution of crystallite size of LS_2_ with temperature is shown in Figure 3. The size of LS_2_ crystals was refined to be about 200 nm at 600°C. Very interestingly, the crystallite size of LS_2_ remained in the same level at the temperature range of 600–700°C where the LS phase co-existed with LS_2_. Right after the disappearance of LS at 700°C, the LS_2_ phase showed a gradual crystal growth.

### Crystallographic change of LS_2_ in the glass

**Figure 4:**
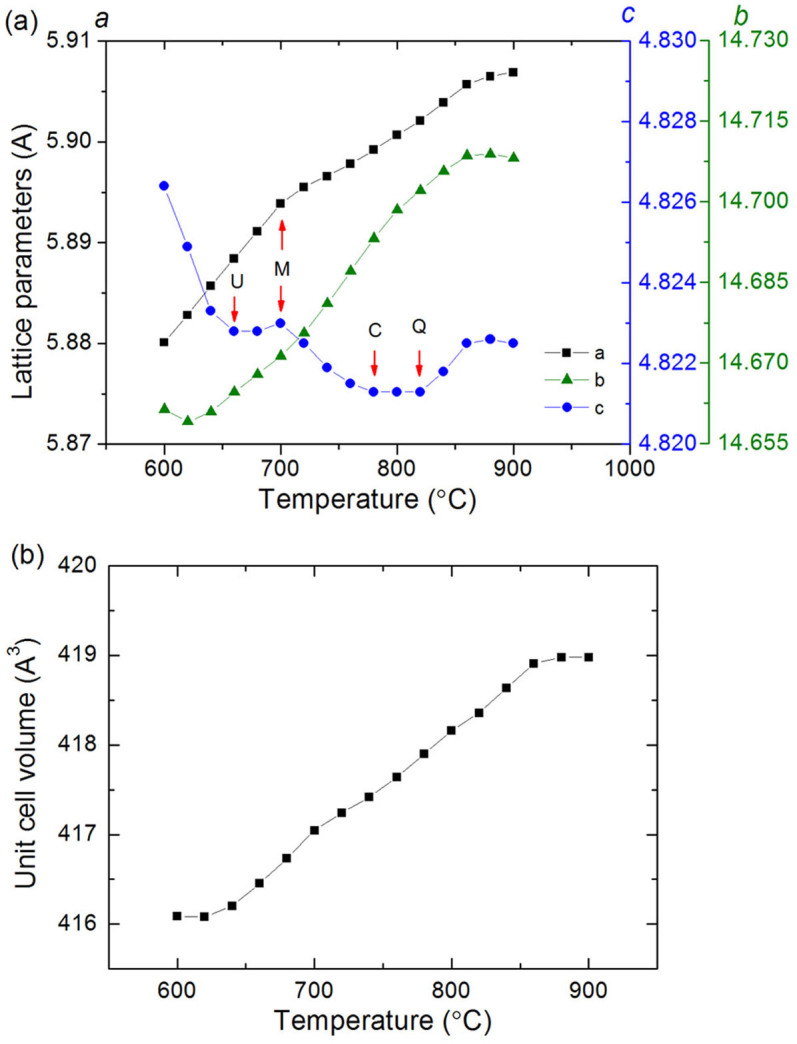
(a) The lattice parameters and (b) unit cell volume of the LS_2_ phase as a function of temperature.

**Figure 5:**
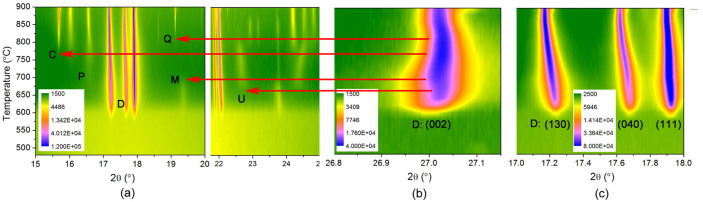
(a) Main peaks of crystalline phases crystallized in the glass as a function of temperature, (b) peak evolution of (002) plane of lithium disilicate phase, (c) peak evolution of its (130), (040), and (111) planes.

The evolution of lattice parameters of LS_2_ as a function of temperature is presented in Figure 4a. A complex trend was observed in the lattice parameter *c* of LS_2_. Specifically, it started with a dramatic decrease at 600–700°C, followed by a slight increase until the LS phase disappeared. The inflection point at 660°C was found to hold a precipitation event of an unidentified intermediate phase. After the completion of LS-to-LS_2_ transformation at 700°C, the parameter *c* showed a reversed trend in that it decreased again until 780°C where a silica phase of β-cristobalite precipitated. From 800°C where another silica phase (β-quartz) crystallized, it began to increase instead. Such a complex trend of crystallographic evolution has been interpreted in the previous findings^5^. The events at transition points of the evolution trend are highlighted by the selected peaks in Figure 5.

The crystallographic *a* and *b* axes do not have similar trend as in the *c* axis, which was observed previously as well^5^. They basically had an increasing trend. As the small amounts of additives (other than SiO_2_, Li_2_O, P_2_O_5_) was not detected to crystallize from the glass matrix, the influence of them on the change of the *c*-axis of LS_2_ is unclear at the time being. The as-measured unit cell volume (*V*) of the LS_2_ phase, which can be calculated by multiplying the parameters, i.e. *V* = *a* × *b* × *c*, shows a near linear increasing trend as a function of temperature (Figure 4b).

## Discussion

It is noteworthy that the LS phase in this glass was not detected by the conventional XRD technique, either *in situ* or *ex situ*^14^. With the laboratory data, the as-investigated glass herein should be regarded as a glass with type III reaction sequence. Then, it could lead to a “LS-free” nucleation mechanism as the LS phase was blindly neglected due to the shortness of laboratory technique. With the aid of the advanced synchrotron radiation, we managed to track the trace phase formation of LS, from a trace amount as low as 0.08 wt% to the maximum fraction of ~2 wt% (Figure 2), which nucleated simultaneously with LS_2_ at 580°C. Correspondingly, the involved mechanism is revised to be that the crystallization of this glass follows the type I sequence^5^.

The formation of limited fraction of LS phase has also been reported by Soares Jr *et al*. during the investigation of crystallization of binary Li_2_O-SiO_2_ glasses^16^. The LS phase coexisted with LS_2_ up to 120 hours at the glass transition temperature, suggesting the same reaction sequence in the glasses involving the formation of trace LS phase.

From the crystallographic aspect, it has been found that the initial evolution trend of the *c* axis of LS_2_ was opposite in the glasses of type I and III sequences, i.e., decreasing and increasing respectively^5^,^6^. Thus, crystallographic interaction within silicates is indicated. The observed mode of crystallographic evolution of LS_2_ in this glass is consistent with that in the glasses of type I sequence^5^,^6^, rather than those of type III sequence. A slight change in the evolution trend of lattice parameter *a* was observed at 700°C (Figure 4a), which probably indicates the effect of the disappearance of LS phase on this crystallographic axis.

From this study and the recent observations^5^,^6^, we believe that a common phenomenon of structural response takes place during the crystallization of lithium disilicate glasses. To be specific, the crystallographic change of LS_2_ in *c* axis was largely affected from a normal increasing trend (if no LS precipitated, i.e., the case in type III sequence) to the opposite with the existence of LS phase (i.e., the cases in types I and II sequences). In addition, the silicon-related phase formation will also affect the evolution trend. The reversed trend has also been observed in other glasses^5^ upon the precipitation of the crystalline phases that share the [O–Si–O] source from glass matrix, such as silicates (LS, LS_2_), silica (CR, QZ, etc.) and other Si-related phases. Unsurprisingly, the unidentified intermediate phases also have an effect on such a trend (Figure 4a), indicating that it may be silicon-containing in composition.

The crystallite size of LS_2_ at the temperature range where LS crystallizes remains at the same level of around 200 nm, and then it gradually increases (Figure 3). A similar trend was reported in other lithium disilicate glasses^7^. This may imply that the existence of LS in the glass not only affects the crystallographic structure of LS_2_ but also hampers its crystal growth capability.

In summary, the state-of-the-art synchrotron radiation enables the detection of the trace phase formation of Li_2_SiO_3_ in a complex lithium disilicate glass by *in situ* X-ray diffraction investigation. The peaks of Li_2_SiO_3_ in very weak intensity was successfully observed at 580–700°C. The kinetics study indicates that the crystallite size of Li_2_Si_2_O_5_ is hampered by the coexistence of LS phase. The crystallographic evolution of Li_2_Si_2_O_5_ showed a complex trend. Particularly in *c* axis, it demonstrates a phenomenon of structural response to other silicon-related phases, such as Li_2_SiO_3_ and silica phases. This phenomenon has been also observed in other glasses, suggesting the generalization of crystallographic interaction phenomenon during glass crystallization.

## Methods

**Figure 6:**
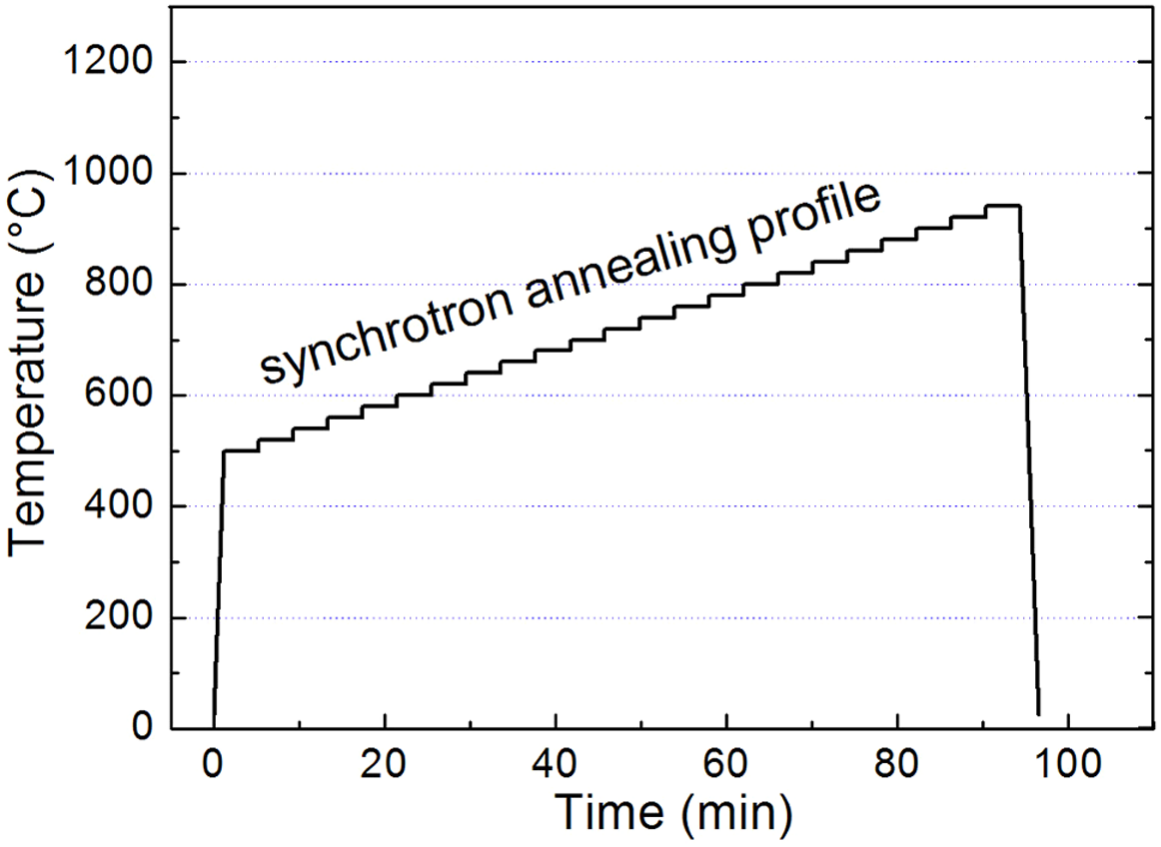
Temperature profile of glass annealing for synchrotron measurements.

The composition of the complex lithium disilicate glass was reported in our previous paper^14^. The main composition of this glass were 63.3SiO_2_, 26.4Li_2_O, 2.6CaO, 1.7P_2_O_5_, 0.9ZrO_2_, 1.3Na_2_O, 1.1K_2_O, 2.7MgO (in mol.%). The glass was melted in a Pt crucible at 1500°C for 3 hrs. The glass samples were mechanically ground to the dimensions of about 5 mm × 5 mm × 0.4 mm. Then they were subjected to synchrotron X-ray diffraction (SXRD) at elevated temperatures on the Powder Diffraction Beamline in the Australian Synchrotron. The energy of the X-ray beam was 11 keV (corresponding wavelength of 1.1273 Å). The detector setup is the same with that described previously^5^,^6^,^7^. The monolithic glass samples were heated up in an Anton Paar HTK-2000 furnace with a platinum (Pt) resistance strip heater at a ramp rate of 400 K·min^−1^. There were 22 holding stages within the temperature range of 500–920°C, with an interval of 20°C (Figure 6), for recording the diffraction patterns. At each stage, X-ray was switched on for 4 min to detect the phases at corresponding temperatures. During the in-situ measurements, no external reference materials were used.

After acquiring the data, the structure refinement was conducted with the MAUD software on the basis of the full-pattern analysis using the Rietveld method^17^. The data sets acquired at 580–900°C were refined sequentially, where a Delf line broadening model was employed and an iterative least-square procedure by minimizing the residual parameters *R_wp_*, *R_B_* and *R_exp_* was adopted. The crystallite size and the microstrain (root mean square, r.m.s. strain, <*ε*^2^>^1/2^) were evaluated using an isotropic size-strain model. Those patterns without the peaks of crystalline phase(s) (i.e., at 500–560°C) and those collected after sample distortion (i.e., at 920°C) were not used for the analyses. During Rietveld analyses, the CIF files ICSD-64980 (Inorganic Crystal Structure Database), ICSD-44095, ICSD-100402, ICSD-280481, and ICSD-79427 were referenced for the profile fitting of β-quartz, β-cristobalite, lithium metasilicate, lithium disilicate, and lithium orthophosphate phase, respectively.

## Author Contributions

S.H., P.C. and W.G. conceived and designed the experiments. S.H. carried out the experiments. S.H. and P.C. analyzed the data. P.C., W.G. and Z.H. supervised the experiments and discussed the results. S.H. wrote the paper with the input from all co-authors.

## References

[b1] Fokin V. M., Zanotto E. D., Yuritsyn N. S. & Schmelzer J. W. P. Homogeneous crystal nucleation in silicate glasses: A 40 years perspective. J. Non-Cryst. Solids 352, 2681–2714 (2006).

[b2] Kemény T. & Šesták J. Comparison of crystallization kinetics determined by isothermal and non-isothermal methods. Thermochim. Acta 110, 113–129 (1987).

[b3] Henderson D. W. Thermal analysis of non-isothermal crystallization kinetics in glass forming liquids. J. Non-Cryst. Solids 30, 301–315 (1979).

[b4] Henderson D. W. Experimental analysis of non-isothermal transformations involving nucleation and growth. J. Therm. Anal. Calorim. 15, 325–331 (1979).

[b5] Huang S., Huang Z., Gao W. & Cao P. Structural Response of Lithium Disilicate in Glass Crystallization. Cryst. Growth Des. 14, 5144–5151 (2014).

[b6] Huang S., Huang Z., Gao W. & Cao P. In Situ High-Temperature Crystallographic Evolution of a Nonstoichiometric Li_2_O·2SiO_2_ Glass. Inorg. Chem. 52, 14188–14195 (2013). 24266416 10.1021/ic402112z

[b7] Huang S., Cao P., Li Y., Huang Z. & Gao W. Nucleation and crystallization kinetics of a multi-component lithium disilicate glass by *in situ* and *real-time* synchrotron X-ray diffraction. Cryst. Growth Des. 13, 4031–4038 (2013).

[b8] von Clausbruch S. C., Schweiger M., Höland W. & Rheinberger V. The effect of P_2_O_5_ on the crystallization and microstructure of glass-ceramics in the SiO_2_-Li_2_O-K_2_O-ZnO-P_2_O_5_ system. J. Non-Cryst. Solids 263–264, 388–394 (2000).

[b9] Höland W., Apel E., van t Hoen C. & Rheinberger V. Studies of crystal phase formations in high-strength lithium disilicate glass-ceramics. J. Non-Cryst. Solids 352, 4041–4050 (2006).

[b10] Hammetter W. F. & Loehman R. E. Crystallization Kinetics of a Complex Lithium Silicate Glass-Ceramic. J. Am. Ceram. Soc. 70, 577–582 (1987).

[b11] Barker M. F., Wang T.-H. & James P. F. Nucleation and growth kinetics of lithium disilicate and lithium metasilicate in lithia-silica glasses. Phys. Chem. Glasses 29, 240–248 (1988).

[b12] Apel E. *et al.* Inventors; Ivoclar Vivadent AG, assignee. Lithium disilicate glass ceramic. United States patent US 7,871,948 B2. Jan 18 2011.

[b13] Höland W. *et al.* Future perspectives of biomaterials for dental restoration. J. Eur. Ceram. Soc. 29, 1291–1297 (2009).

[b14] Huang S., Cao P., Wang C., Huang Z. & Gao W. Fabrication of a high-strength lithium disilicate glass-ceramic in a complex glass system. J. Asian Ceram. Soc. 1, 46–52 (2013).

[b15] Bischoff C., Eckert H., Apel E., Rheinberger V. M. & Holand W. Phase evolution in lithium disilicate glass-ceramics based on non-stoichiometric compositions of a multi-component system: structural studies by ^29^Si single and double resonance solid state NMR. Phys. Chem. Chem. Phys. 13, 4540–4551 (2011). 21270993 10.1039/c0cp01440k

[b16] Soares Jr P. C., Zanotto E. D., Fokin V. M. & Jain H. TEM and XRD study of early crystallization of lithium disilicate glasses. J. Non-Cryst. Solids 331, 217–227 (2003).

[b17] Lutterotti L., Matthies S. & Wenk H.-R. MAUD: a friendly Java program for material analysis using diffraction. IUCr: Newsletter of the CPD 21, 14–15 (1999).

